# Supplementary material for: Patterns of Intron Gain and Loss in Fungi
Source: PLoS Biol. 2004 Nov 30;2(12):e422. doi: 10.1371/journal.pbio.0020422 (PMC532390; doi:10.1371/journal.pbio.0020422)
Supplement: Table S1 — Also available at http://genes.mit.edu/NielsenEtAl/. (4.3 MB ZIP). [file pbio.0020422.st001.zip › NielsenEtAl/html/126.html]

AN1776.1.NCU05645.1.MG03758.1.FG02192.1


```
 CLUSTAL W (1.82) Multiple Sequence Alignments - Introns Inserted


Sequence 1: NCU05645.1	284 aa
Sequence 2: MG03758.1	283 aa
Sequence 3: FG02192.1	270 aa
Sequence 4: AN1776.1	303 aa
Alignment Length: 314 aa
Number Identitical Residues: 157 aa
Alignment Score (without introns) 7263


MG03758.1 	MAAEQRKLLE1QLMGG~-------GIAARPAQVSLTDSKVCRSYVAGTCPHDLFTNTRQD
NCU05645.1	MAAEQRKLLE1QLMGG~-------GISSRSAQLSLTDPKVCRSYLVGTCPHDLFTNTKQD
FG02192.1 	MAAEQRKLLE~QLMGA~-------SSTSRAAQLSLTDPKVCRSYLAGTCPHDLFTNTKQD
AN1776.1  	MAAEQRKLLE~QLMGA1DQLIGIPGAPSRNAQLSITDPKVCRSYLVGTCPHDLFTNTKQD
          	********** ****. ..  . .. .:* **:*:**.******:.***********:**

MG03758.1 	LGPCPKVHSEALKAEYEALPEKERQKMGFEYDYMRDLQKYIDECNRRIDAAQRRLEKTPD
NCU05645.1	LGPCSRVHSEALKAEYEALPEQEKKKYGFDYDYMRDLQKYIDECNRRIDAAQRRLEKTPD
FG02192.1 	IGPCPKVHNEGLKTEYEALSDREKQKYGFEYDYMRDLQKYIDDCNRRIDAAQRRLEKTPD
AN1776.1  	LGPCPKVHSEGLKTEYETASAADKAKWGFEYDYMRDMQKYIDDCDRRIESAQRRLEKTPD
          	:***.:**.*.**:***: .  :: * **:******:*****:*:***::**********

MG03758.1 	EIRQTNVL0LRTISDLQNSINCGLVEVEILGESGQVTLALDEYVRVRQNQAAKAEREKEL
NCU05645.1	EIRQTNVL~LKAISDLGTTIATGLLEVEILGELGEVGRAYDELFRVRQAQAAKQEKEREL
FG02192.1 	EIRQTSVL0LKSISELTDSINNGLLEVEVLGSMGEVSRAQDELFRVRQASQSKADREKEL
AN1776.1  	EIRQTNNL0LKQISDLTQTINTGLLEVSVLGETGSVAQALNELHKIRTAKHQKETCEREL
          	*****. * *: **:*  :*  **:**.:**. *.*  * :*  ::*  .  *   *:**

MG03758.1 	RALADTGGPSGHQKLQVCDVCGAYLSRLDNDRRLADHFYGKMHLGYAQMRKTYDAFPKEL
NCU05645.1	KALSDTSGPSGHQKLQVCDVCGAYLSRLDNDRRLADHFFGKMHLGYAQMRKTYDAFPKEM
FG02192.1 	KALSDTSGPSGHQKLQVCDVCGAYLSRLDNDRRLADHFYGKMHLGYAQMRKTYDAFPKEM
AN1776.1  	KNLQDTSGPSGHQKLQVCDVCGAYLSRLDNDRRLADHFFGKMHMGYSDMRKGWKKLHEEL
          	: * **.*******************************:****:**::*** :. : :*:

MG03758.1 	RSR----GGGGGRMPLDDHG--QSGPGGYDDGWKGPRGP-~------------RAGGYGG
NCU05645.1	RGRQRPMGMGGGGD--DDMGGVPTGPGGRHDDWGRGRGP-~------------RSG---G
FG02192.1 	KGR--------SRAPMDDDG-----PG-----MGGPRGP-~------------RGSGGYR
AN1776.1  	KGR-----PPPMRHHDEDDG------GGWGGRSGGGRGPS2YLLHKTKSPSVQRISYLLG
          	:.*             :* *      *.  .     ***:     .:.:.: .* .    

MG03758.1 	GGGGGRGGRGRRW-----
NCU05645.1	GGYRGRGPRRGGW-----
FG02192.1 	SGRGGRGYRGG-W-----
AN1776.1  	GGYVLQAVTNHVYEWLST
          	.*   :.     :.  ::
```
